# Dataset of compression after impact testing on carbon fiber reinforced plastic laminates

**DOI:** 10.1016/j.dib.2025.111509

**Published:** 2025-03-22

**Authors:** Saki Hasebe, Ryo Higuchi, Tomohiro Yokozeki, Shin-ichi Takeda

**Affiliations:** a Department of Aeronautics and Astronautics, The University of Tokyo, 7-3-1 Hongo, Bunkyo-ku, Tokyo 113-8656 Japan; b Aviation Technology Directorate, Japan Aerospace Exploration Agency (JAXA), 6-13-1 Osawa, Mitaka-shi, Tokyo 181-0015, Japan

**Keywords:** Thermoset CFRP, Laminates, Barely Visible Impact Damage, Compression after impact strength, Machine learning

## Abstract

This dataset covers the data obtained from the compression after impact (CAI) tests. Before the CAI tests, the low-velocity impact (LVI) testing was conducted under various experimental conditions (layup, impactor shape, and impact energy) to simulate various foreign object impacts on actual structures. For the CAI tests, both the specimens used in the LVI tests and undamaged specimens were utilized to calculate the strength reduction rate. This dataset includes a test condition list and raw and processed data: 1. LVI test conditions, 2. Specimen size, 3. Specimen appearance after the CAI tests, 4. Raw data obtained from the data logger during the testing, and 5. CAI strength. This dataset is created to seek a way to predict CAI strength using information on damage in CFRP specimens and the experimental condition. The data are helpful for researchers and engineers who are involved in the impact behavior or residual characteristics of CFRP and artificial intelligence.

Specifications Table

**Table:** 

Subject	Material Characterization
Specific subject area	Compression after impact characteristics on composite materials, and Machine learning
Type of data	Table, Image, Chart. Raw, Processed.
Data collection	Before the CAI tests, the specimen sizes were measured with callipers. The applied load was measured using a compression testing machine during the CAI tests, and the strain was measured using strain gauges. After the experiment, the surface damage of the test specimens was photographed to record their condition*.*
Data source location	Data were obtained from the Yokozeki lab, department of Aeronautics and Astronautics, The University of Tokyo, Tokyo, Japan, and Aviation Technology Directorate, Japan Aerospace Exploration Agency, Tokyo, Japan.
Data accessibility	Repository name: Mendeley Data Data identification number: 10.17632/8scdmfdcfb.3 Direct URL to data: https://data.mendeley.com/datasets/8scdmfdcfb/3
Related research article	S. Hasebe, R. Higuchi, T. Yokozeki, S. Takeda, Prediction of compression after impact strength from surface profile of low-velocity impact damaged CFRP laminates using machine learning, Composites Part A, 108560, 2024. doi: 10.1016/j.compositesa.2024.108560 [1].

## 1 Value of the Data

• This dataset is valuable because it includes raw data from CAI tests conducted after LVI tests under various impact conditions, including different layups (including hard and soft laminates designed to sustain axial and shear loads), impactor shapes, and impact energies to simulate foreign object impacts on actual structures. While standardized testing protocols exist, real-world impacts occur under uncontrolled conditions. By considering practical applications, this dataset provides a broader range of test data, making it particularly useful.
• This dataset is expected to be useful for researchers and engineers involved with structures using CFRPs, because reproducing real-world conditions in laboratory tests requires extensive testing, which is often costly and time-consuming. By utilizing this dataset, they can analyze impact behavior and failure mechanisms under diverse conditions while minimizing the need for additional experiments.
• The dataset is expected to benefit AI researchers and engineers, particularly in machine learning. Conventional LVI and CAI test data often include limited samples (e.g. around 50), posting challenges for machine learning applications. This dataset comprises over 400 samples, significantly improving its suitability for such applications and expanding opportunities for AI research.

## 2 Background

CFRP structures, such as those in aircraft, can suffer damage from foreign object collisions, leading to external and internal damages like matrix cracks, fiber breakage, and delamination. Even Barely Visible Impact Damage (BVID) can contribute to a reduction in residual strength.

Previous studies have provided valuable insights into CFRP impact damage, including data on stacking sequences, materials, energy levels [2,3], non-standard boundary conditions [4], and CFRP repair methods [5]. These contributions are vital in advancing understanding of these phenomena.

This study conducted LVI and CAI testing to consider the diversity of impact conditions in practical environments, collecting data by varying laminate configuration, energy, and impactor shape. Methods to predict impactor shape and internal damage from BVID-related external damage were examined through LVI tests [6,7], and CAI strength prediction based on external and internal damage was validated through CAI tests [1]. The data has been compiled into an editable dataset, making it accessible to both structural engineers and AI researchers. This dataset is expected to bridge the gap between research and practical applications.

## 3 Data Description

The data is all stored in Mendeley data [8], and the folder structures are as follows:

1. Low velocity impact testing condition
  This folder includes the LVI test conditions, and the LVI damage data for each specimen. The impact condition includes three parameters, layup, impactor shape and impact energy. Regarding the LVI damage, the list has two characteristic parameters, projected delamination area, and dent depth.
2. Specimen size
  This contains the specimen size data, which was measured before the CAI testing. The list includes height, width and thickness data of each specimen.
3. Specimen image
  In this folder, the images of each specimen after the CAI testing are stored.
4. Compression after impact testing raw data
  This folder includes the raw data which is obtained from the data logger during the CAI testing. Each csv file has 2 data columns obtained directly from the compression testing machine, Extension [V], and Load [V], and 4 data columns from the strain gauges, Strain-FL [µm], Strain-FR [µm], Strain-BL [µm], Strain-BR [µm], which F, B, L, and R represents front, back, left and right, respectively.
5. Compression after impact strength
  In ``Compression after impact strength'' folder, the maximum stress value and the strength reduction rate list of each testing are stored. The calculation details are described in the next section.

The specimen name is the same as the previous datasets of the authors, which include the more detailed LVI damage data [[9], [10], [11], [12], [13], [14], [15]].

## 4 Experimental Design, Materials and Methods

1. Specimen preparation before low-velocity impact test [6,15]
  The material used in this study was a carbon/epoxy unidirectional system T800S/3900-2B, which was produced by Toray Industries Inc. All panels were fabricated using an autoclave under conditions of a 180 °C curing temperature. While the material properties were not assessed in this research, prior studies conducted extensive experiments to obtain them [16]. Table 1 presents an overview of the mechanical properties.

**Table 1:** Material properties [16].

Material properties	Values
Longitudinal tensile Young's modulus *E_11t_*	153 GPa
Longitudinal compressive Young's modulus *E_11 c_*	132.6 GPa
Transverse Young's modulus *E_22_*	8.0 GPa
Poisson's ratio *ν_12_*	0.34
Shear modulus *G_12_*	4.03 GPa
Longitudinal tensile strength *X_t_*	3100 MPa
Longitudinal compressive strength *X_c_*	719.4 MPa
Interlaminar fracture toughness (mode I) *G_IC_*	0.54 N/mm
Interlaminar fracture toughness (mode II) *G_IIC_*	1.64 N/mm
  A composite material cutting machine (AC-500CF, AC-400CF) manufactured by Maruto Testing Machine Company was used to cut the specimens (Fig. 1). First, the AC-500CF was used to divide large plates (450 mm × 450 mm) into sections with a side ratio of 2:3. Then, the AC-400CF was used to cut specimens with a side length of 80 mm (Fig. 2). Totally 422 specimens were prepared for the LVI testing.

**Fig. 1:**
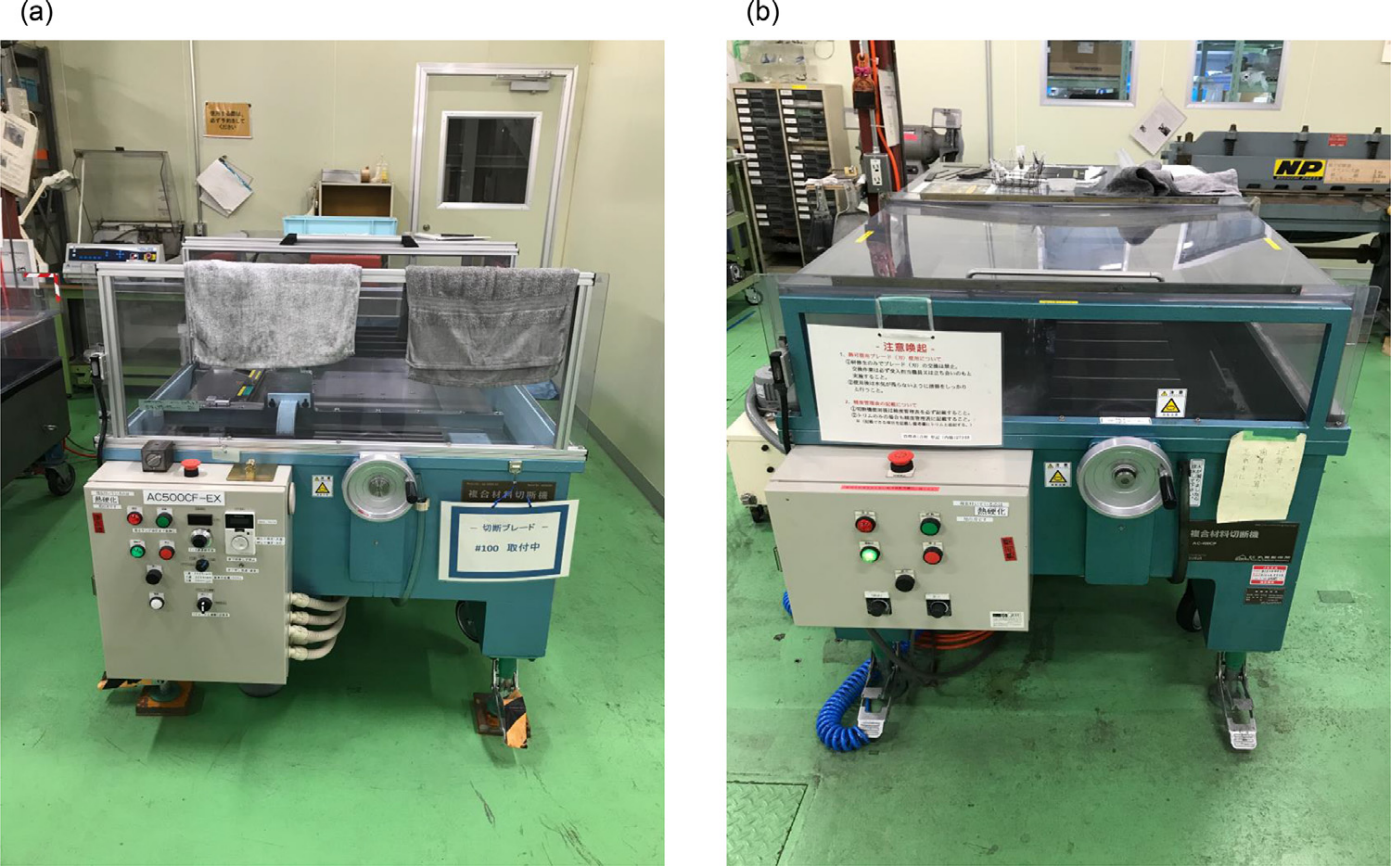
Cutting machine (a)AC-500CF, (b) AC-400CF.

**Fig. 2:**
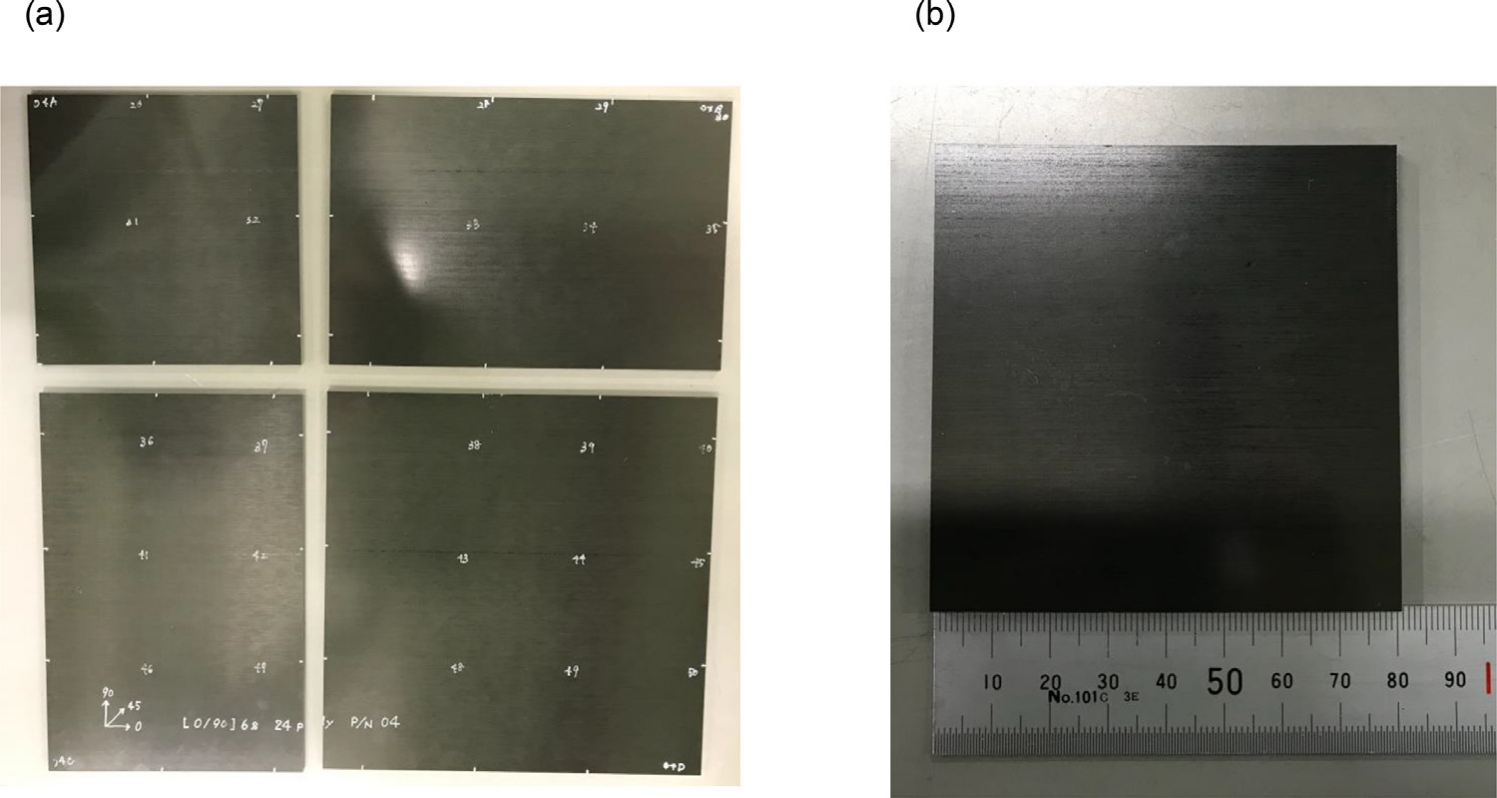
Specimens (a) segmented into four sections, (b) used for the test.
2. Low-velocity impact test [6,15]
  The LVI testing was conducted using a drop weight impact testing machine (CEAST 9350, Instron) (Fig. 3). Generally, drop weight impact tests conform to ASTM D7136 standards [17]. However, in this case, the specimen size was changed from 150 [mm] by 100 [mm] to 80 [mm] by 80 [mm] to increase the number of samples.

**Fig. 3:**
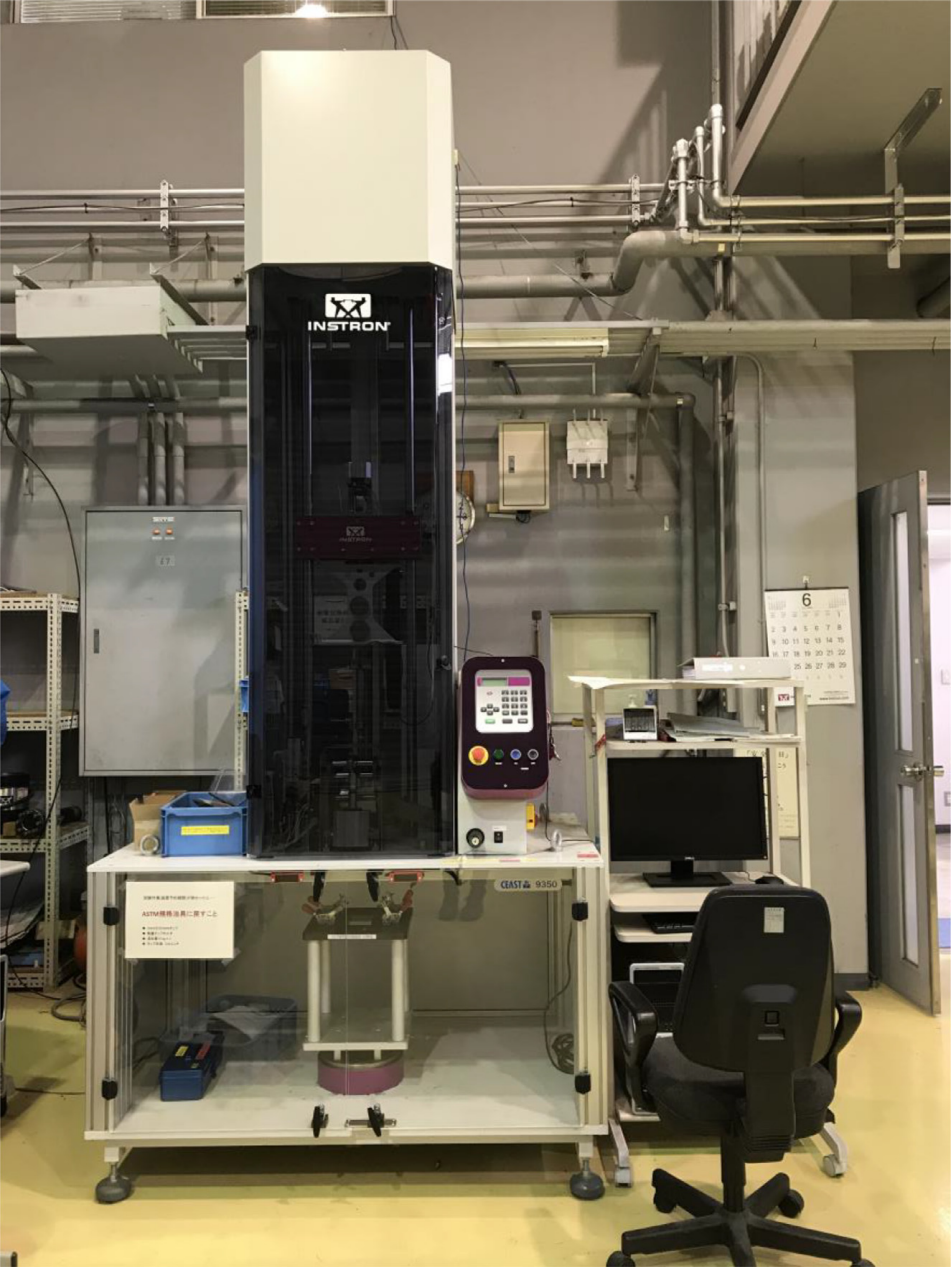
Drop tower impact system CEAST 9350.
  In order to simulate actual foreign object impact, the three impact conditions were changed (Table 2). The first parameter considered in this study was the stacking sequence. Three types of laminates were examined: cross-ply laminates (c8 ([0/90]_2s_), c16 ([0/90]_4s_), and c24 ([0/90]_6s_)), quasi-isotropic laminates (q8 ([45/0/−45/90]_s_), q16 ([45/0/−45/90]_2s_), and q24 ([45/0/−45/90]_3s_)), hard laminates (r0([0/45/0/90/0/-45/0/45/0/-45]_s_), and soft laminates (r45([45/-45/0/45/-45/90/45/-45/45/-45]_s_)). The average ply thickness was approximately 0.1875 mm. The second parameter was the impactor shape. Considering that foreign objects with different shapes can collide on the structures in an actual situation, this experiment was conducted using six different impactors: ``HemiA,'' ``HemiB,'' ``HemiC,'' ``Coni60,'' ``Coni120,'' and ``Flat,'' as illustrated in Fig. 4. The HemiA, HemiB, and HemiC impactors have hemispherical heads with diameters of 12.7 mm, 25.4 mm, and 31.8 mm, respectively. The Coni60 and Coni120 impactors are conical, featuring vertical angles of 60° and 120°, respectively. Lastly, the Flat impactor has a diameter of 15.9 mm and a flat striking surface. These impactors are made of medium carbon steel S45C. The third parameter was the impact energy level. Because the specimen dimensions were smaller than those specified in ASTM D7136 [17] and conical impactors were used in this study, the experimental setup could cause severe damage to the specimens. To mitigate this, the energy levels were reduced to around less than half of the standard 6.7 J/mm, with values of 4.4, 3.35, 2.7, 2.2, 1.6, and 1.1 J/mm. One to three specimens were prepared for each impact condition. See the “1_Low velocity impact testing condition” folder for the impact condition of each specimen.

**Table 2:** Low velocity impact testing conditions.

Layup	Stacking sequence	No. of plies	Average thickness [mm]	Impact energy [J/mm]	Impactor shape
c8	[0/90]_2s_	8	1.5	1.6, 2.2, 2.7, 3.35, 4.4	HemiA, HemiB, HemiC, Coni60, Coni120, Flat
c16	[0/90]_4s_	16	3.1	1.6, 2.2, 2.7, 3.35, 4.4	HemiA, HemiB, HemiC, Coni60, Coni120, Flat
c24	[0/90]_6s_	24	4.6	1.1, 1.6, 2.2, 2.7, 3.35, 4.4	HemiA, HemiB, HemiC, Coni60, Coni120, Flat
q8	[45/0/−45/90]_s_	8	1.5	1.6, 2.2, 2.7, 3.35, 4.4	HemiA, HemiB, Coni60, Coni120, Flat
q16	[45/0/−45/90]_2s_	16	3.1	1.6, 2.2, 2.7, 3.35, 4.4	HemiA, HemiB, Coni60, Coni120, Flat
q24	[45/0/−45/90]_3s_	24	4.6	1.6, 2.2, 2.7, 3.35, 4.4	HemiA, HemiB, Coni60, Coni120, Flat
r0	[0/45/0/90/0/-45/0/45/0/-45]_s_	20	3.8	2.2, 3.35, 4.4	HemiA, HemiB, Coni60, Coni120, Flat
r45	[45/-45/0/45/-45/90/45/-45/45/-45]_s_	20	3.8	2.2, 3.35, 4.4	HemiA, HemiB, Coni60, Coni120, Flat

**Fig. 4:**
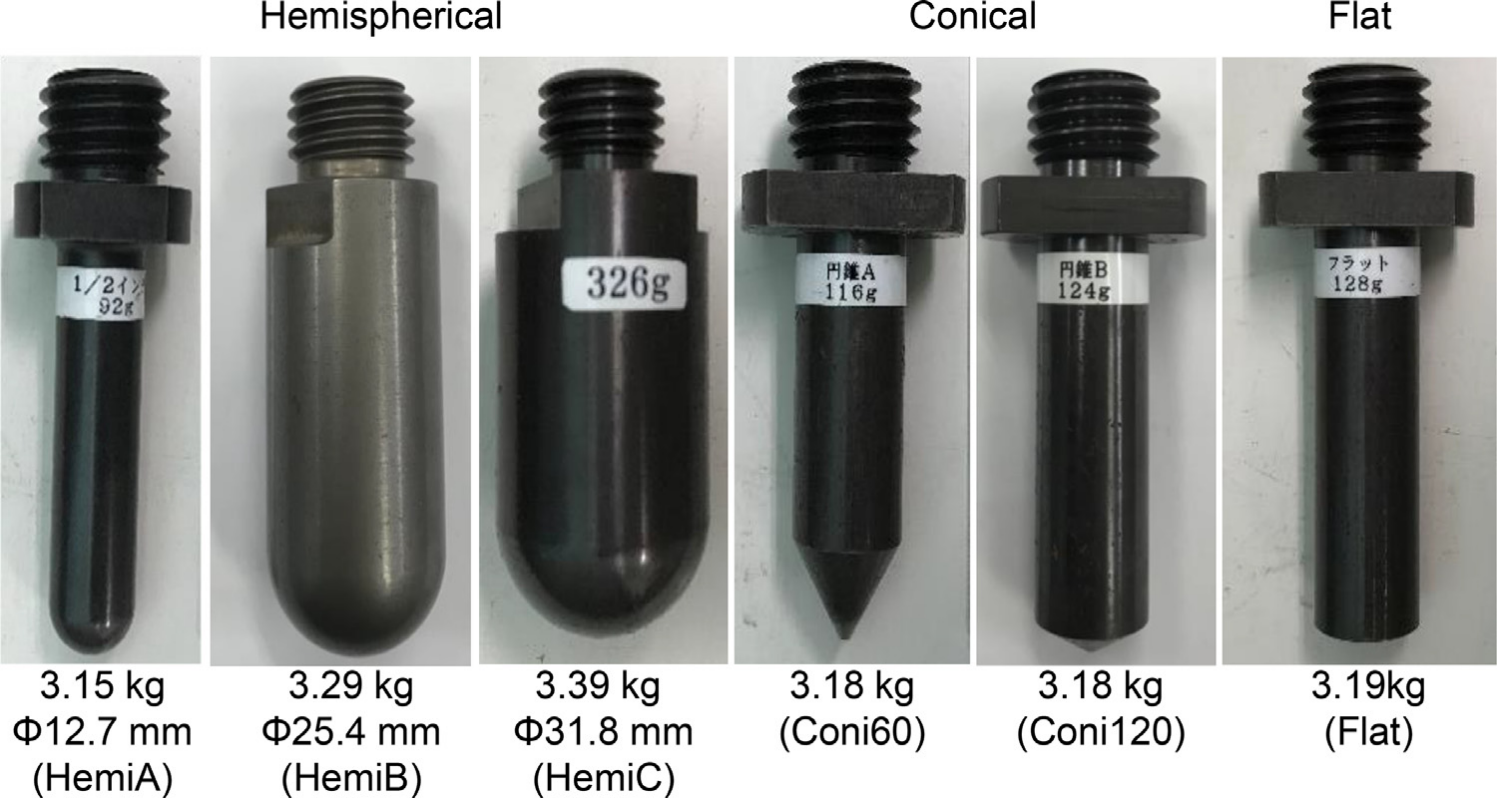
Impactor shape.
  The specimens were clamped using two plates, made of SUS304 stainless steel, with a 60 [mm] by 60 [mm] cutout and bolted with a torque of 5 [Nm] (Fig. 5). To reproduce these data, put the specimen on the center of fixing tools. Otherwise, the boundary condition lacks uniformity. The load cell capacity was 40 [kN], and data was acquired at a frequency of 200[kHz] or 1[MHz]. The drop height ℎ [m] is calculated by the software based on the impact energy *E* [J], impactor mass *m* [kg], and gravitational acceleration *g* [m/s²]:
  (1) h=E/mg

**Fig. 5:**
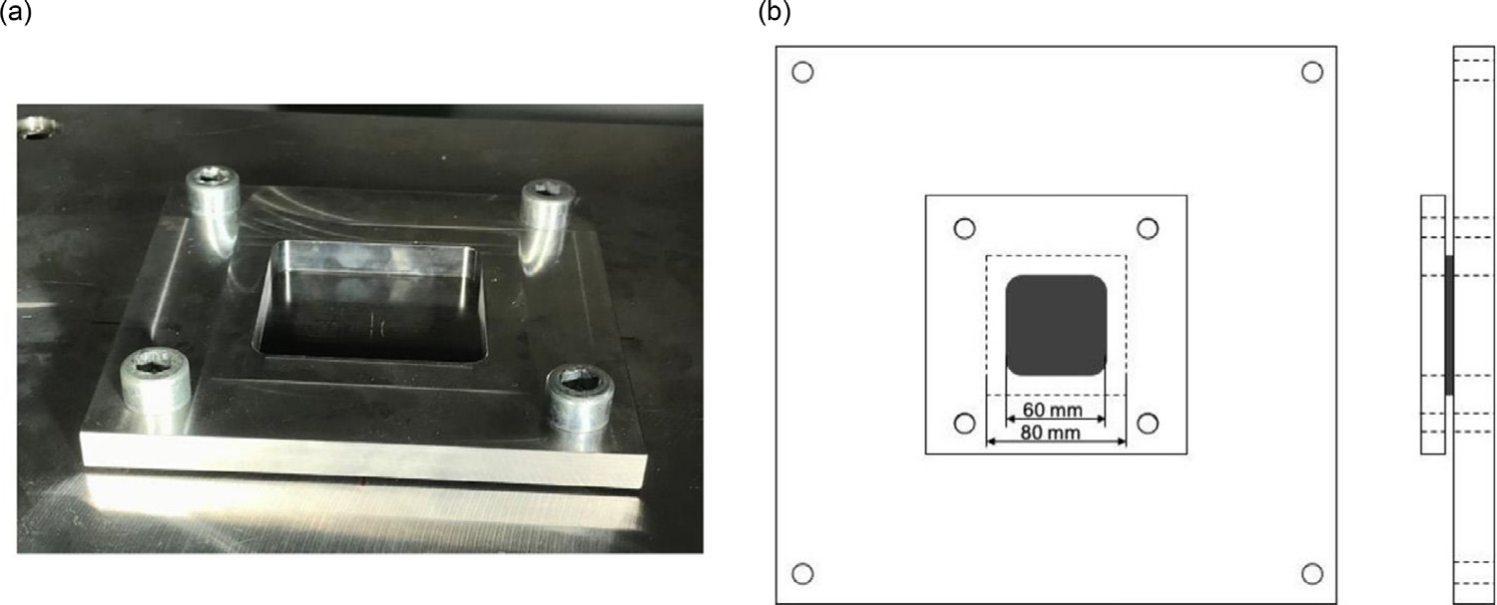
Specimen fixture: (a) image and (b) diagram.
3. Post analysis of low velocity impact test [6,15]
  To measure the surface profile, the software integrated with the VR-5000 (Keyence Corporation) was utilized (Fig. 6). The analysis begins by defining a reference plane, representing the undeformed intact surface, using mean-square fitting based on the four corners of each specimen. Because all four edges were fixed during the LVI test, the relative positions of these corners are assumed to remain unchanged before and after testing. Once the reference plane is established, the software calculates the depth at each point on the specimen. This software considers the bulging direction positive, and the indentation depth corresponds to the minimum surface depth.

**Fig. 6:**
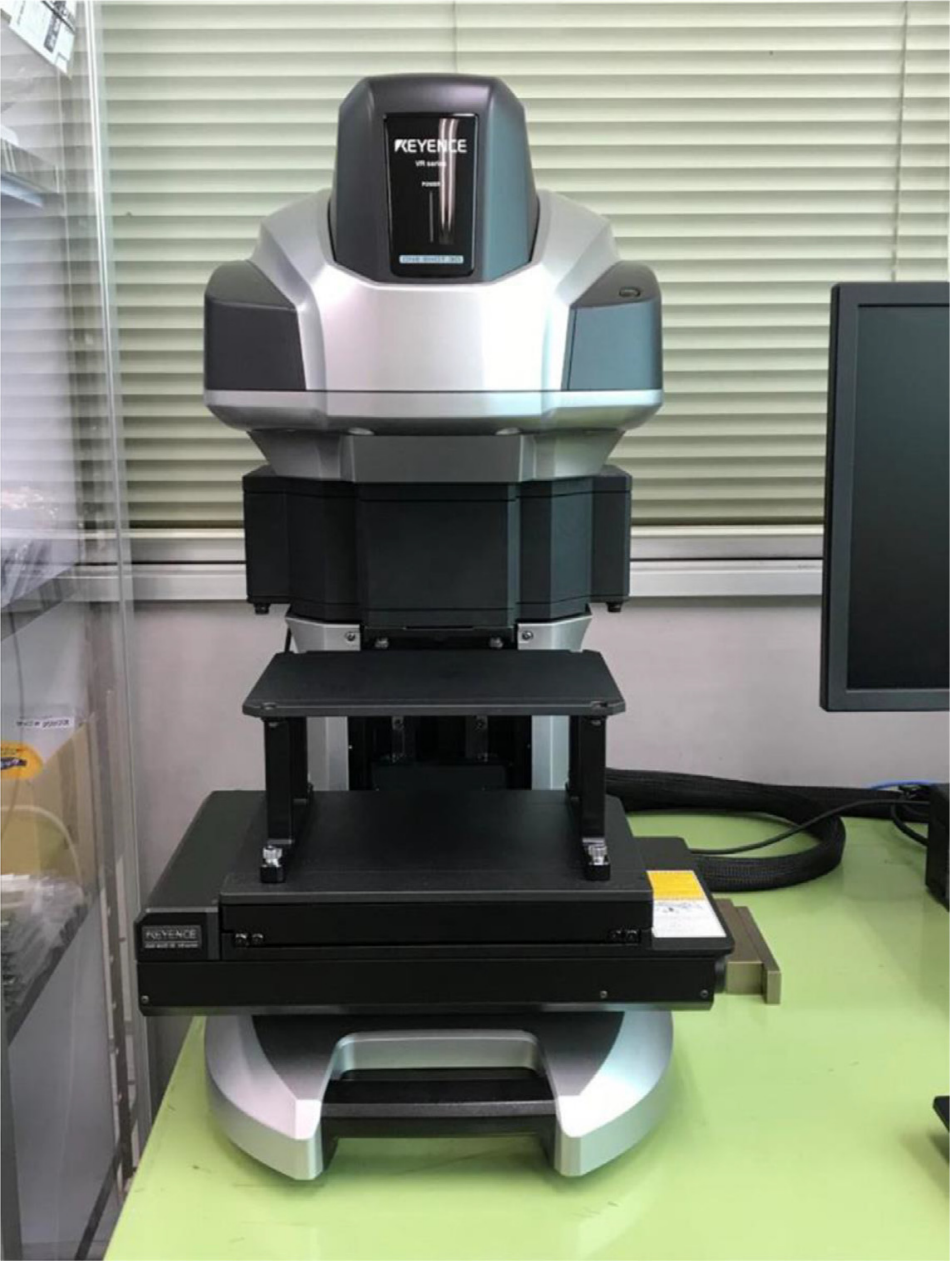
Wide-area 3D measurement system VR-5000.
  To assess the internal damage of the impacted specimen, ultrasound C-scanning was performed (Fig. 7). The probe frequency used in this measurement was 3.2 [MHz]. Because the software does not support exporting raw data in a non-proprietary format, screenshots were captured and saved as image data. The delamination area was measured from the C-scanning images using the image processing and analysis software ImageJ.

**Fig. 7:**
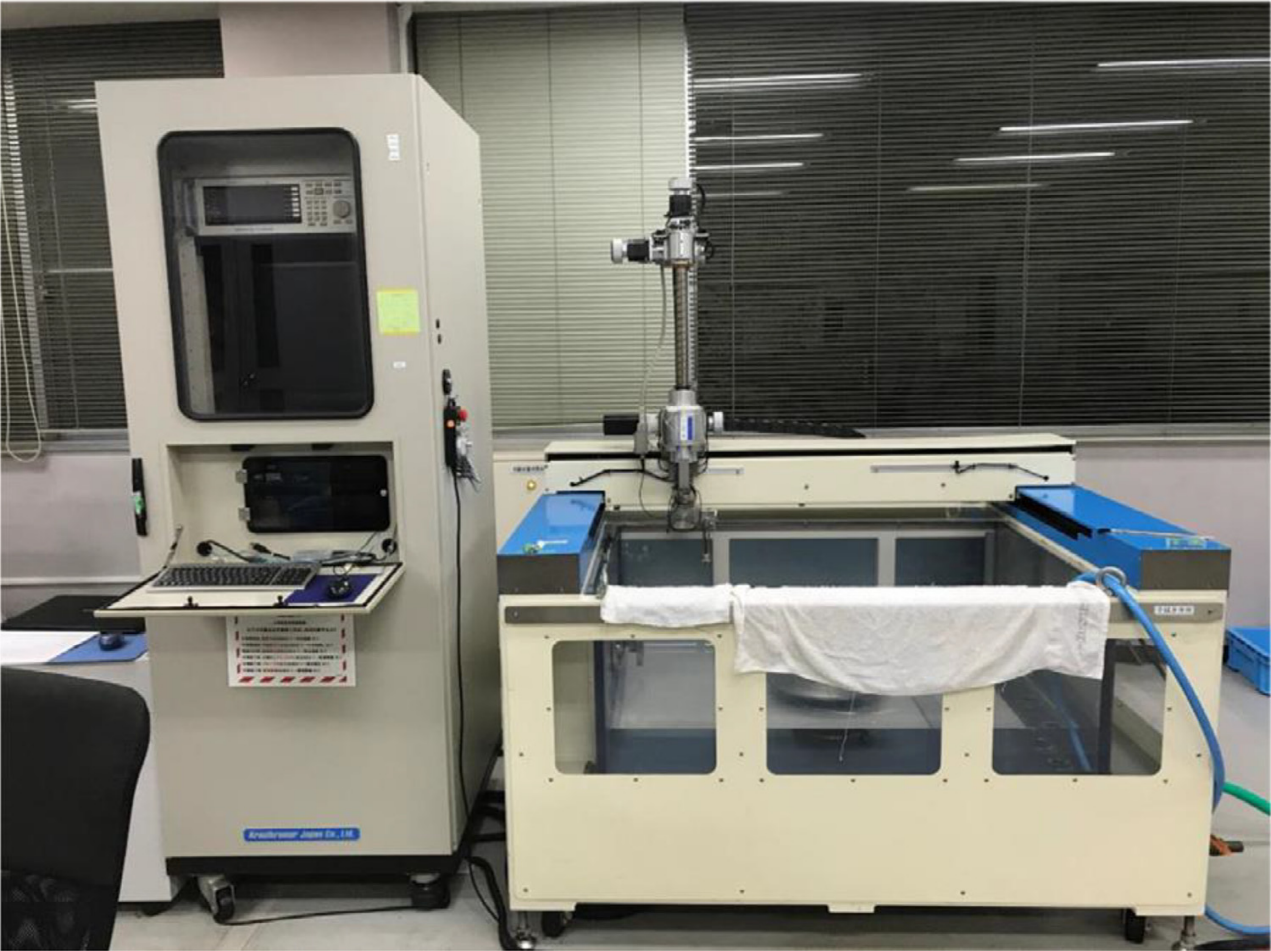
Ultrasound C-scanning system HIS3.
  For example, Fig. 8 depicts the images of data obtained from surface profile measurement and ultrasound C-scanning for two specimens (r0-1 and r45-1). These specimens are impacted under the same conditions (impactor = HemiA, impact energy = 3.35 J/mm), except the layup.

**Fig. 8:**
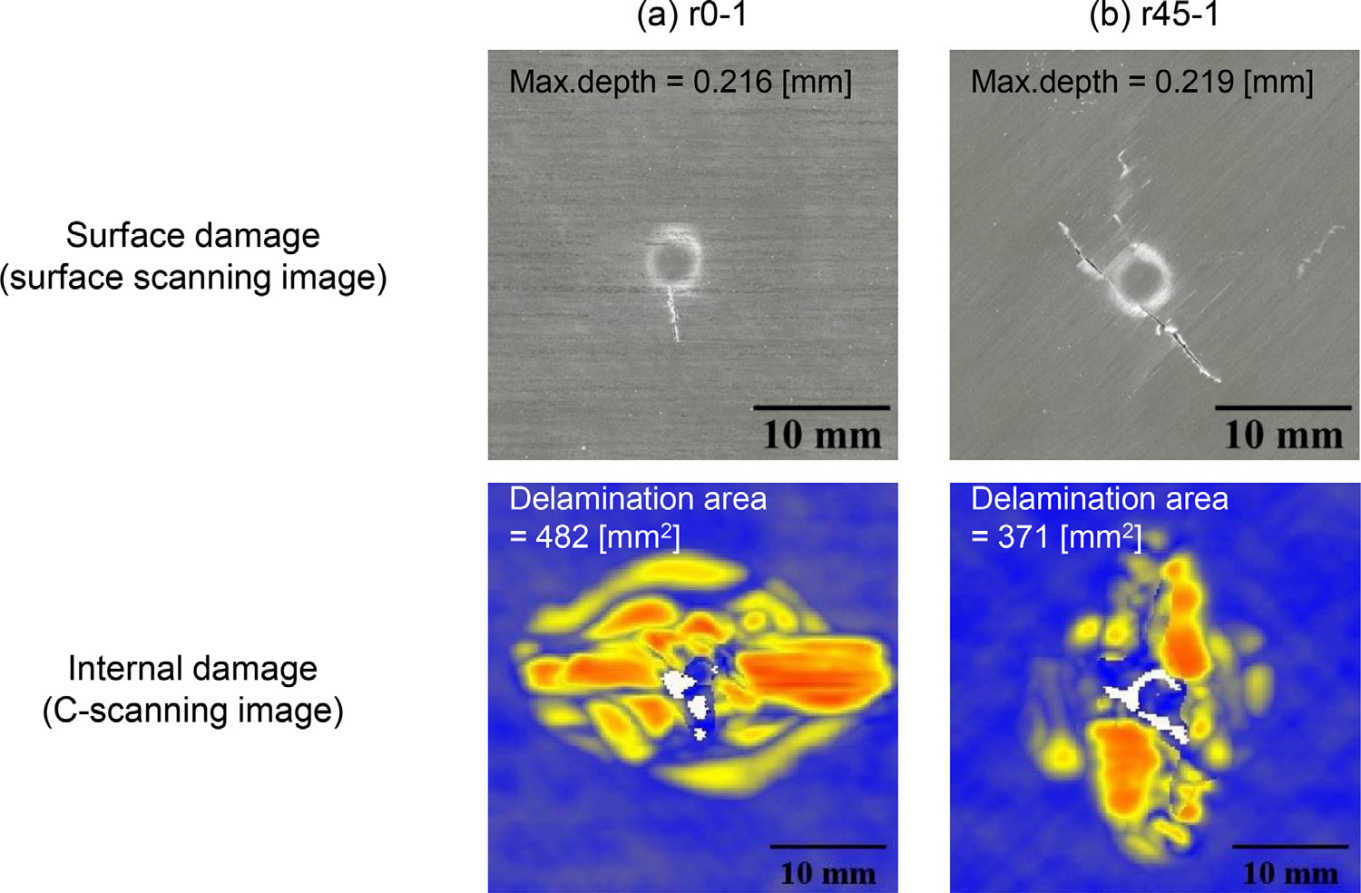
Surface and internal damage of specimens after LVI testing (a) r0-1 (layup=r0, impactor=HemiA, impact energy=3.35 J/mm), (b) r45-1 (layup=r45, impactor=HemiA, impact energy=3.35J/mm).
4. Specimen preparation before compression after impact test
  All specimens for the CAI testing were reshaped to 80 [mm] in the 0° direction by 50 [mm] in the 90° direction by cutting off 15 [mm] from each side [18]. A Caliper was used to measure the specimen size: length, width and thickness. The total of 446 specimens include 26 non-damaged and 420 LVI damaged ones.
5. Compression after impact test
  The CAI testing was conducted using a universal testing machine (5885H, Instron), referring to the standards in the reference section of JIS K7089 [18]. The jig was made of SUS630 stainless steel, and bolted with a torque of 7 [Nm]. The load was applied in displacement control mode using a load cell with a capacity of 250 [kN], with a crosshead speed of 1.0 [mm/min], and the data was acquired at a rate of 50 [Hz]. Two gauges (KFGS-3-120-C1-11L1M2R, KYOWA) were installed on both the front and back of each specimen (Fig. 9). The conversion factors for displacement, load, and strain gauge factor are 1 [mm/V], 25 [kN/V], and 2.11 % ± 1.0 %, respectively.

**Fig. 9:**
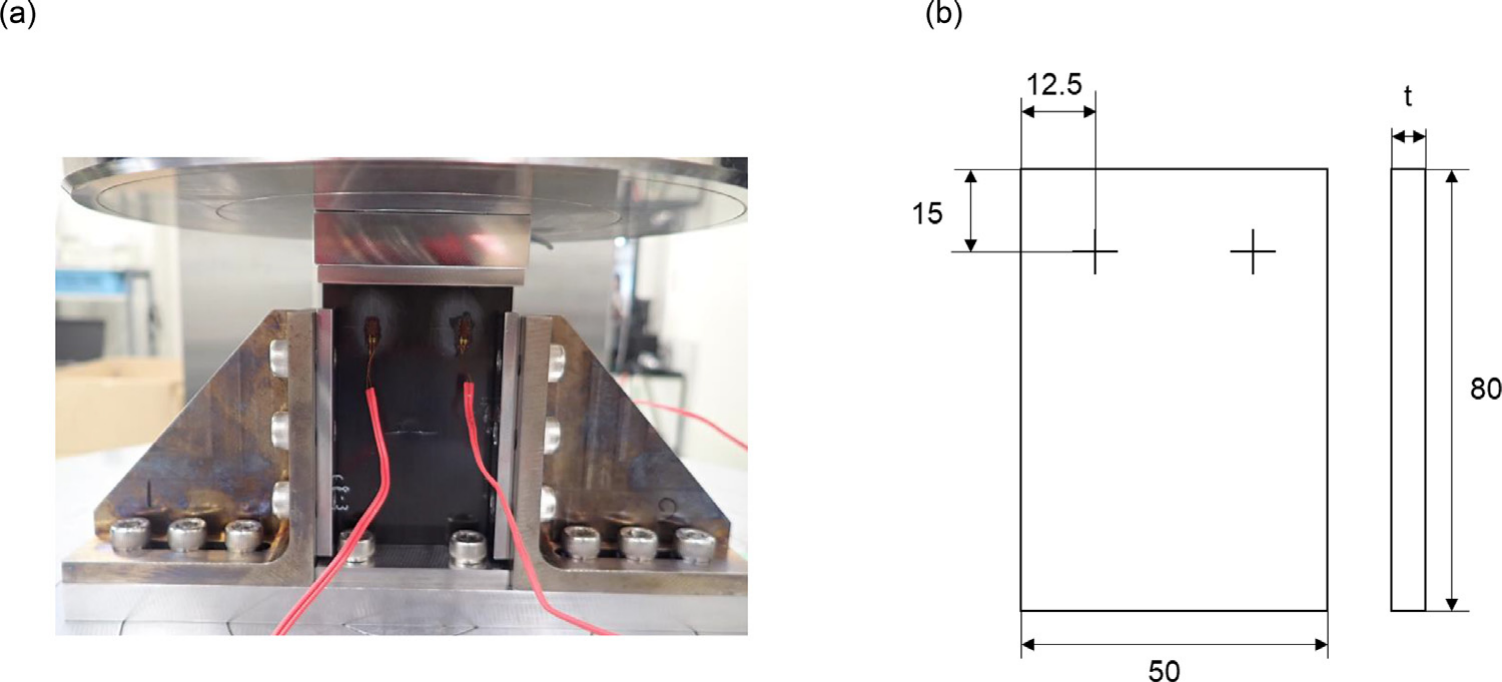
Compression after impact testing setup (a) image, (b) strain gage locations [mm].
6. Post analysis of compression after impact test
  Soon after the testing, the surface damage of the specimens was photographed with a usual camera in order to record the damage condition.
  Stress was calculated from the experimental data. Load *F_calc_*[kN] is calculated by multiplying the Load *F_raw_*[V] obtained from the compression testing machine by a conversion factor of 25[kN/V].
  (2) $$F_{calc}\left[\text{kN}\right]=F_{raw}\left[V\right]\times 25\left[\text{kN}/V\right]$$
  The stress *σ*[MPa] is then calculated by dividing the obtained load by the cross-sectional area of the specimen *A* [mm^2^].
  (3) $$\sigma \left[\text{MPa}\right]=F_{calc}\left[\text{kN}\right]*10^{3}/A\left[mm^{2}\right]$$
  The maximum absolute value of this σ represents the CAI strength. Also, The CAI strength reduction rate is defined as the ratio of the CAI strength of a damaged specimen to that of an intact specimen as follows:
  (4) $$CAI\;strength\;reduction\;rate\left[\%\right]=\sigma_{max,damaged}\left[\text{MPa}\right]/\sigma_{max,intact}\left[\text{MPa}\right]$$
  Fig. 10 depicts the damage observed in the r0-1 and r45-1 specimens, confirming that both specimens failed in the central region near the impact damage.

**Fig 10:**
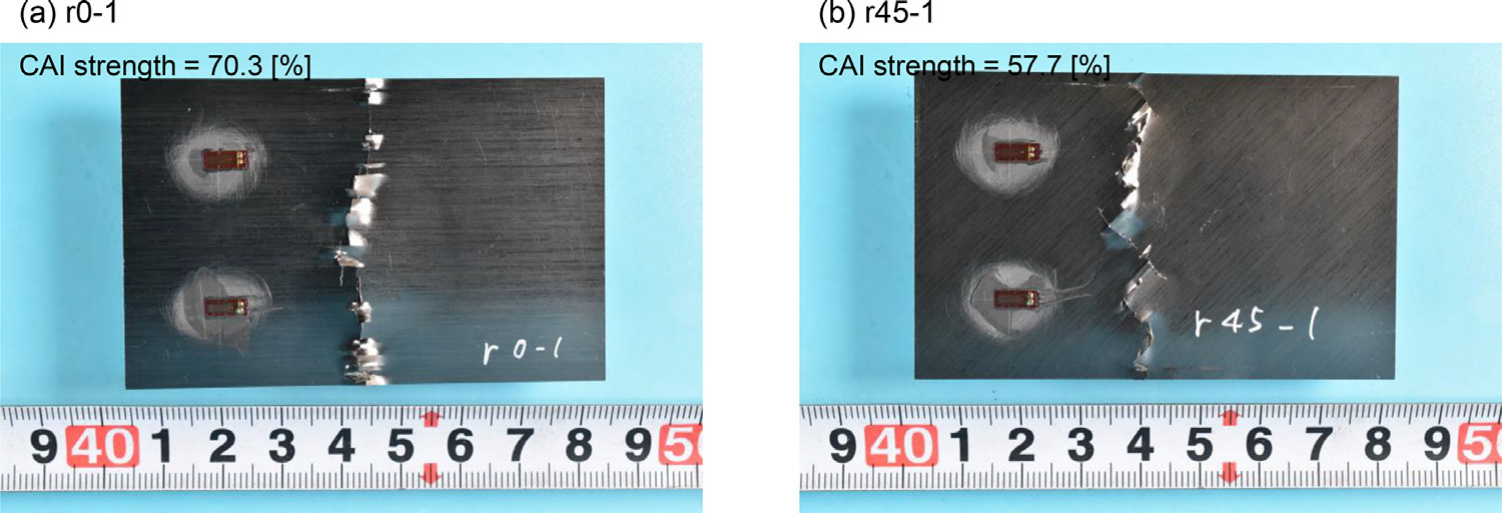
Surface damage of specimens after CAI testing(a) r0-1 (layup=r0, impactor=HemiA, impact energy=3.35 J/mm), (b) r45-1 (layup=r45, impactor=HemiA, impact energy= 3.35J/mm).

## Limitations

None

## Ethics Statement

The authors have read and follow the ethical requirements for publication in Data in Brief, and the current work does not involve human subjects, animal experiments, or any data collected from social media platforms.

## CRediT authorship contribution statement

**Saki Hasebe:** Methodology, Investigation, Writing – original draft. **Ryo Higuchi:** Conceptualization, Writing – review & editing. **Tomohiro Yokozeki:** Supervision. **Shin-ichi Takeda:** Validation, Supervision.

## Data Availability

Mendeley DataDatasets on CFRP specimens subjected to compression after impact tests (Original data).
